# The Role of Deubiquitinating Enzymes in Synaptic Function and Nervous System Diseases

**DOI:** 10.1155/2012/892749

**Published:** 2012-12-18

**Authors:** Jennifer R. Kowalski, Peter Juo

**Affiliations:** ^1^Department of Biological Sciences, Butler University, 4600 Sunset Avenue, Indianapolis, IN 46208, USA; ^2^Department of Molecular Physiology and Pharmacology, Tufts University School of Medicine, 150 Harrison Avenue, Boston, MA 02111, USA

## Abstract

Posttranslational modification of proteins by ubiquitin has emerged as a critical regulator of synapse development and function. Ubiquitination is a reversible modification mediated by the concerted action of a large number of specific ubiquitin ligases and ubiquitin proteases, called deubiquitinating enzymes (DUBs). The balance of activity of these enzymes determines the localization, function, and stability of target proteins. While some DUBs counter the action of specific ubiquitin ligases by removing ubiquitin and editing ubiquitin chains, other DUBs function more generally to maintain the cellular pool of free ubiquitin monomers. The importance of DUB function at the synapse is underscored by the association of specific mutations in DUB genes with several neurological disorders. Over the last decade, although much research has led to the identification and characterization of many ubiquitin ligases at the synapse, our knowledge of the relevant DUBs that act at the synapse has lagged. This review is focused on highlighting our current understanding of DUBs that regulate synaptic function and the diseases that result from dysfunction of these DUBs.

## 1. Introduction to the Ubiquitin Signaling System

Over the past decade, the ubiquitin signaling system has become a well-established regulator of neuronal biology [1–4]. In neurons, ubiquitin controls diverse cellular processes including cell fate determination, cell survival, neurite outgrowth and morphogenesis, synapse development, and synaptic function [1, 3–5]. Misregulation of the ubiquitin system is linked to numerous neurological and neurodegenerative disorders [3–8]. Despite the identification and characterization of several ubiquitin pathway enzymes that are involved in these processes, much remains to be elucidated regarding the function, regulation, and substrates of the majority of ubiquitin enzymes in neurons and, in particular, at synapses. Below, we will provide a general overview of ubiquitin system biology and its impact on neuronal function, followed by a more focused analysis of the known roles of deubiquitinating enzymes (DUBs) in controlling synaptic activity.

**Figure 1:**
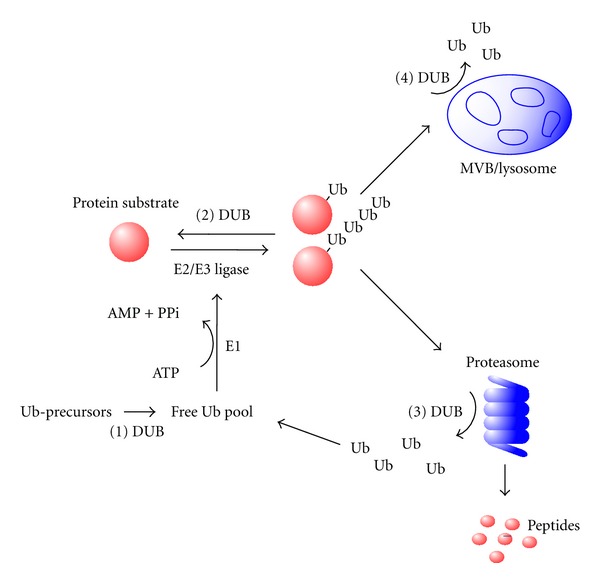
The ubiquitin signaling system. Ubiquitin is activated by an ubiquitin-activating enzyme (E1), transferred to a ubiquitin-conjugating enzyme (E2) and, with the help of an ubiquitin ligase (E3), is covalently attached to lysine residues on specific substrates [2]. In addition to altering protein function or subcellular localization, monoubiquitination can target proteins for endocytosis and degradation in the multivesicular body (MVB)/lysosome pathway. Ubiquitin can form polyubiquitin chains via seven different lysine residues. Recent studies indicate that K63 chains are required to target substrates for degradation in the MVB/lysosome pathway [21]. Polyubiquitin chains using K48 linkages consisting of four or more ubiquitin molecules target proteins for degradation in the 26S proteasome. Recent studies indicate that polyubiquitin chains using other linkages like K11, K27, and K29 can also target proteins for proteasomal degradation [9, 10, 22]. DUBs function at multiple steps in the ubiquitin system: (1) DUBs are required to generate free Ub monomers from ubiquitin precursors, (2) DUBs counter the action of ubiquitin ligases, (3) DUBs function at the proteasome to edit ubiquitin chains, to remove ubiquitin prior to substrate degradation in the proteasome, and to recycle monomeric ubiquitin, and (4) DUBs function at the MVB to promote recycling of monomeric ubiquitin by removing ubiquitin prior to internalization of substrates into the MVB [15, 23, 24].

Protein ubiquitination is a critical posttranslational modification that occurs in all eukaryotes where it serves to regulate the stability, activity, and/or localization of both soluble and transmembrane proteins in diverse cell types. Ubiquitin itself is a 76 amino acid polypeptide that is covalently added to lysine residues in target proteins by the activity of a three-step enzymatic pathway consisting of a ubiquitin-activating enzyme (E1) that forms a thiol-ester intermediate with the C-terminal glycine residue of a ubiquitin monomer, a ubiquitin-conjugating enzyme (E2) to which the activated ubiquitin is transferred, and a ubiquitin ligase (E3), which along with the E2 enzyme, conjugates ubiquitin to specific substrates (Figure 1) [2]. Covalent attachment of a single ubiquitin to the *ε*-amino group of a lysine residue in substrate proteins (monoubiquitination) can regulate their activity, ability to interact with other proteins, subcellular localization or trafficking. Alternatively, several ubiquitin molecules may be covalently linked together to form ubiquitin chains (polyubiquitination). Ubiquitin itself contains seven lysine residues (K6, K11, K27, K29, K33, K48, and K63), and recent studies suggest that all seven lysines and the amino terminus of ubiquitin can be utilized to form a variety of branched or linear chains that are thought to determine the ultimate cellular fate of the ubiquitinated protein [9, 10].

One major function of ubiquitin is to target proteins for degradation in either the proteasome or lysosome (Figure 1). Substrates containing polyubiquitin chains (such as K48-linked chains) of at least four ubiquitin moieties are typically recognized by proteins containing specific ubiquitin-binding domains that facilitate delivery of the ubiquitinated proteins to the 26S proteasome for degradation. In contrast, monoubiquitination or K63-linked polyubiquitination of transmembrane proteins often serves as a signal for their endocytosis and/or sorting to the multivesicular body (MVB) for degradation in the lysosome [11–14].

Like most posttranslational modifications, ubiquitination is reversible; DUBs counterbalance ubiquitin ligase activity by removing ubiquitin from target proteins (Figure 1) [15–18]. DUBs play a critical role at the proteasome where they are involved in editing ubiquitin chains and removing and recycling ubiquitin prior to substrate degradation in the proteasome. DUBs also regulate protein targeting and degradation in the lysosome [13, 15]. The levels of transmembrane proteins at the cell surface can be regulated by endocytosis followed by either degradation in the lysosome or recycling back to the plasma membrane. Ubiquitinated transmembrane receptors are recognized by the Endosomal Sorting Complex Required for Transport (ESCRT) complex which targets the receptors to the multivesicular body (MVB). Subsequent fusion of the MVB with the lysosome results in receptor degradation [13, 19]. DUBs regulate transmembrane receptor degradation in the MVB/lysosome pathway via three mechanisms: (1) preventing receptor degradation by directly removing ubiquitin from the protein and thus facilitating receptor recycling back to the cell surface, (2) promoting receptor degradation by deubiquitinating and stabilizing the ESCRT complex components responsible for targeting ubiquitinated receptors to the MVB, and (3) promoting receptor degradation and ubiquitin recycling by deubiquitinating the receptor immediately prior to its internalization into the MVB [13, 15, 20].

## 2. Ubiquitin in Synaptic Function

The human genome encodes an estimated 500–600 E3 ubiquitin ligases and about 100 DUBs [15, 17, 25]. The specific substrates and cellular functions of the vast majority of these enzymes in the nervous system are unknown. The first evidence that ubiquitination was important for synaptic function came from work investigating mechanisms of synaptic plasticity in the marine mollusk, *Aplysia*, which identified the DUB Ap-Uch as a critical regulator of long-term facilitation during the gill withdrawal reflex [26]. Subsequent studies in cultured mammalian neurons demonstrated that ubiquitination of synaptic proteins was dynamic and could be directly controlled by both acute and chronic changes in synaptic activity [27, 28]. A series of studies in flies, worms, and mice have now identified specific roles for several ubiquitin ligases and DUBs in controlling synapse development and function (see following reviews: [1, 3–5, 29]). The growing list of ubiquitin system components and target proteins at synapses underscores the importance of the ubiquitin system in synapse biology [1, 3–5]. Furthermore, the accumulation of ubiquitin-conjugated proteins in aggregates and inclusion bodies in neurons in neurodegenerative diseases [7] and the association of mutations in specific ubiquitin ligases and DUBs with several neurological disorders, such as Angelman's syndrome, Parkinson's Disease and ataxia [5, 6, 30–32], emphasize the need for a more complete understanding of the role of ubiquitin in the nervous system.

Although initial studies focused on elucidating the functions and substrate specificities of E3 ubiquitin ligases, a plethora of recent reports have described roles for DUBs in diverse cellular processes, ranging from membrane receptor trafficking and ubiquitin recycling to effects on transcription, chromatin structure, and DNA repair [15, 23]. The DUBs encoded by the human genome can be grouped into five classes based on their sequence homology within the catalytic domain. These include 4 classes of cysteine proteases: the Ubiquitin C-terminal Hydrolases (UCHs; 4 members), the Ubiquitin Specific Proteases (USPs; 57 members), the Machado Joseph Disease proteases (MJD; 4 members), and the Otubain proteases (OTU; 13 members). The fifth class is composed of the JAB1/MPN/Mov34 enzymes (JAMM; 8 members), which are metalloproteases [17].

Several excellent reviews have discussed the structure and function of DUBs in a wide range of cellular processes in detail [15, 16, 23, 25], including a recent review focused on the role of DUBs in the nervous system [24]. Here, we describe the role of the few DUBs that have been shown to specifically control synaptic function (Table 1), and if known, discuss how their dysfunction contributes to neurological disorders.

## 3. DUBs Controlling Ubiquitin Homeostasis at the Synapse

### 3.1. UCH-L1/Ap-Uch: A Regulator of Monomeric Ubiquitin That Controls Synapse Structure and Function

The expression of carboxyl-terminal hydrolase, UCH-L1, is almost exclusively restricted to the brain, testes, and ovaries [33, 73]. In neurons, UCH-L1 is highly expressed and represents 1-2% of total soluble brain proteins [74]. Several lines of evidence link UCH-L1 to multiple neurodegenerative disorders in both mice and humans, underscoring the importance of UCH-L1 in neuronal function. First, together with ubiquitin, UCH-L1 is enriched in the protein aggregates and inclusion bodies associated with Parkinson's and Alzheimer's Diseases [34–36]. Second, a specific familial mutation in *UCH-L1 *is associated with Parkinson's Disease in humans, and transgenic mice expressing the same UCH-L1 mutation exhibit a loss of dopaminergic neurons [35, 37, 38]. Third, a different spontaneous mutation in UCH-L1 in mice results in gracile axonal dystrophy (*gad*), which is characterized by an accumulation of ubiquitinated protein aggregates in neurons, axonal degeneration in the spinal gracile tract, and late-onset progressive ataxia [33, 39–41].

Biochemically, UCH-L1 has been shown to possess several functions. UCH-L1 can increase levels of monomeric ubiquitin in neurons by binding and stabilizing ubiquitin monomers and by deubiquitinating ubiquitin precursors [42, 43]. UCH-L1 dimers have also been reported to possess ubiquitin ligase activity, which can regulate the degradation of *α*-synuclein [44]. Finally, UCH-L1 can be farnesylated, and this membrane-associated form of the DUB can promote the accumulation and toxicity of *α*-synuclein [45].

Work in *Aplysia* led to the initial identification of Ap-Uch as the first DUB known to regulate synaptic activity [26]. Ap-Uch shares similarities with both mammalian UCH-L1 and UCH-L3. Sequence comparison indicates that Ap-Uch is more similar to UCH-L3. However, the expression pattern of Ap-Uch is more closely related to UCH-L1, because unlike UCH-L3 which is broadly expressed in many tissues, Ap-Uch is exclusively expressed in the nervous system [26]. Ap-Uch is an immediate early gene induced by the transcription factor CREB during long-term facilitation (LTF), a form of plasticity in *Aplysia* [26]. In this system, the Ap-Uch protein associates with the proteasome where it promotes the recycling of ubiquitin and the degradation of substrates, such as the regulatory (R) subunit of PKA, which is involved in inhibiting LTF. Inhibition of Ap-Uch activity, by delivering blocking antibodies or antisense olignucleotides specifically into sensory neurons, inhibits LTF in *Aplysia*, suggesting a presynaptic site of action [26]. Studies in mammals indicate that *UCH-L3-*deficient mice have defects in working memory, while *UCH-L1 *mutant *gad* mice exhibit an accumulation of ubiquitinated proteins [33, 46, 51]. Together, these studies suggest that proper control of ubiquitin levels in neurons is critical for normal synaptic function and that defects in the DUBs involved in this process impact synaptic plasticity.

Additional studies revealed further mechanisms by which UCH-L1 contributes to activity-dependent control of synaptic function at glutamatergic synapses. Specifically, NMDA treatment of cultured hippocampal neurons resulted in increased activation of UCH-L1 and increased levels of monomeric ubiquitin [47]. Conversely, pharmacological inhibition of UCH-L1 activity resulted in decreased levels of monomeric ubiquitin and decreased rates of proteasome-mediated degradation. These effects on the ubiquitin system were accompanied by several defects in synapse structure including decreased spine density, increased spine size, and increased accumulation of pre- and postsynaptic proteins. In addition, inhibition of UCH-L1 resulted in abnormal pre- and postsynaptic terminals at the ultrastructural level, including excessive numbers of presynaptic vesicles and enlarged terminals and aberrant mitochondria and vacuoles [47]. The defects in synapse structure can be attributed to the ability of UCH-L1 to maintain monomeric ubiquitin levels because overexpression of ubiquitin restored normal synaptic structure to UCH-L1 deficient neurons [47]. Similar results were observed in UCH-L1 knockout mice, which exhibit impaired spontaneous and evoked synaptic activity at neuromuscular junctions [48]. These functional defects were correlated with a reduction in synaptic vesicle number, a concomitant increase in aberrant tubulovesicular structures in axon terminals and, ultimately, de-nervation of the muscle [48]. Thus, defects in synaptic transmission may underlie the peripheral neurodegeneration observed in UCH-L1-deficient animals.

A second role for UCH-L1 in neurodegeneration was observed in a mouse model of Alzheimer's Disease (AD) pathogenesis [49]. Levels of soluble UCH-L1 were previously shown to be downregulated in the brains of AD patients, where it is found associated with neurofibrillary tangles [50]. *APP/PSI* transgenic mice, which are a model of AD, possess reduced UCH-L1 activity in the brain, and this correlates with significant decreases in the levels of both monomeric ubiquitin and LTP [49]. Similar results are seen in hippocampal slice cultures treated with A*β*_42_ oligomers. In both cases, however, these phenotypes are ameliorated by overexpression of a catalytically functional UCH-L1 hydrolase [49]. Consistent with the findings in *Aplysia* [26], the ability of UCH-L1 expression, and specifically its hydrolase activity, to reduce the defects in synaptic plasticity in AD mouse models are due to its ability to reduce levels of the R subunit of PKA back to wild type levels [49]. Interestingly, several recent studies also demonstrated the ability of UCH-L1 to regulate the abundance of *β*-site amyloid precursor protein (*β*-APP) cleaving enzyme 1 (BACE1), the secretase enzyme critical for the generation of A*β* peptides [75, 76]. Specifically, UCH-L1 appears to increase lysosomal degradation of BACE1, as inhibition of UCH-L1 caused a significant increase in BACE1 protein levels in several cell types, and loss of *UCH-L1* gene function in *gad* mice significantly increased levels of endogenous BACE1, C99, and A*β* peptides [75, 76]. While the specificity of these effects is unclear, enhancement of UCH-L1 activity may be a promising approach for the treatment of AD. The importance of UCH-L1 in regulating synaptic plasticity and its association with several neurological disorders emphasizes the need to identify relevant substrates of this enzyme and to understand how UCH-L1 activity is regulated in neurons.

### 3.2. Identification of DUBs Associated with Synaptic Proteasomes: USP5, USP7, USP13, USP14, UCHL5/UCH37

The holoenzyme proteasome is a more than 2.5 MDa complex comprised of a core particle containing 28 subunits (the 20S complex) and a regulatory particle containing 19 subunits (the 19S complex) in yeast [57]. Although it was originally thought that the proteasome was a fairly static degradation complex, many recent studies have suggested that the composition of the proteasome is surprisingly dynamic and may differ depending on the subcellular location, the specific cellular conditions, or the cell type where it is expressed [57]. Moreover, evidence for proteasome regulation during synaptic plasticity, including the rapid recruitment of proteasomes into dendrites in response to synaptic stimulation, suggests that synaptic proteasomes may have different subunit compositions or mechanisms of regulation [77–79]. Thus, Tai et al. decided to take a proteomics approach to define the subunit composition of synaptic proteasomes [52]. Using mass spectrometry to analyze the composition of both cytosolic and synaptic proteasomes from adult rat cortical hippocampal neurons, they identified five proteasome-associated DUBs that copurify with synaptic, as well as cytosolic, 26S proteasomes: USP5, USP7, USP13, USP14/Ubp6, and UCH37/UCH-L5 [52]. They hypothesize that these five DUBs work in conjunction with the constitutively-associated proteasomal subunit and JAMM metallo-protease DUB, Rpn11 (also known as POH1/PSMD14), to trim and remove ubiquitin chains prior to substrate degradation. With the exception of USP14, the mechanisms, effects, and modes of regulation of these proteasome-associated DUBs in synaptic function have not yet been investigated.

### 3.3. USP14: A Proteasome-Associated DUB Involved in Ataxia

USP14 (known as Ubp6 in yeast) is perhaps the second most studied DUB involved in synapse development and function. USP14 is one of three DUBs, together with UCH37 and Rpn11/Poh1, known to be associated with the 19S regulatory component of the proteasome [57–59, 80, 81]. Association of USP14/Ubp6 with the proteasome via its UBL domain stimulates the DUB's catalytic activity several hundredfold [59, 60, 80, 82]. USP14/Ubp6 has several functions at the proteasome. These include inhibition of proteasome-mediated degradation by both trimming of ubiquitin chains conjugated to substrates and by a noncatalytic mechanism [82, 83], regulation of gate opening of the core particle [84], and maintenance of cellular levels of free ubiquitin [58–61, 82]. In yeast and mammals, loss of USP14/Ubp6 results in increased degradation of ubiquitin and decreased levels of monomeric Ub, suggesting that one function of USP14 is to recycle ubiquitin at the proteasome [58, 59, 61, 62].

Most of what we know about USP14 in synapse development and function comes from analysis of the ataxia (*ax* ^*J*^) mutant mouse. *ax* ^*J*^ mice exhibit severe tremors at 2-3 weeks of age, extensive muscle wasting and paralysis in the hind limbs, and ultimately death between 6 and 10 weeks of age [63, 64]. Positional mapping revealed that the *ax* ^*J*^ mouse contains a spontaneous mutation in *Usp14* that results in reduced mRNA and undetectable levels of the full-length USP14 protein [58, 60, 64]. Alternative splicing of *Usp14 *results in a full-length isoform and a short isoform missing the UBL domain required for proteasome association; interestingly, the *ax* ^*J*^ mouse expresses normal levels of the short isoform [60], suggesting that loss of the long form of USP14 is responsible for the defects observed in the *ax* ^*J*^ mouse.

Phenotypic analysis of *ax* ^*J*^ mice identified several defects in synaptic transmission in both the peripheral and central nervous systems. At the neuromuscular junction (NMJ), *ax* ^*J*^ mice exhibit defects in spontaneous and evoked synaptic transmission. Specifically, *ax* ^*J*^ mice have reduced frequencies of spontaneous miniature end-plate potentials (mEPPs) and reduced evoked end-plate currents (EPCs), as well as a corresponding decrease in quantal content [64]. This reduced release was hypothesized to result in a homeostatic increase in postsynaptic responsiveness indicated by the increased mEPP amplitudes observed in the *ax* ^*J*^ mice [64]. These results suggested that *ax* ^*J*^ mice have a presynaptic defect in neurotransmitter release at the NMJ. A recent study supports these findings by showing that *ax* ^*J*^ mice also exhibit reduced paired-pulse facilitation (PPF) at the NMJ and a frequency-dependent increase in run down after high frequency stimulation; these authors thus proposed that *ax* ^*J*^ mice have defects in SV recycling or recruitment under conditions of extreme activity [65].

In addition to these peripheral synaptic defects, *ax* ^*J*^ mice also exhibit defects in synaptic plasticity at central synapses [64]. Recordings of CA3-CA1 synapses in the hippocampus revealed that *ax* ^*J*^ mice have reduced paired-pulse facilitation (PPF) and posttetanic potentiation (PTP) [64, 66]. In contrast, long-term potentiation and the maintenance of long-term depression appeared normal in *ax* ^*J*^ mice [64, 66]. Together, the synaptic transmission defects in *ax* ^*J*^ mice at the NMJ and in the hippocampus suggest that Usp14 plays an important role in regulating synaptic plasticity.

The defects in the *ax* ^*J*^ mice are most likely due to aberrant neuronal and synaptic development [64]. The major cellular phenotype of these mice appears to be a reduction in monomeric (i.e., free) ubiquitin levels by about 35% in both neuronal and nonneuronal cells [58]. Larger reductions in both monomeric and conjugated ubiquitin were observed in synaptosomes from *ax* ^*J*^ mice, perhaps because synapses are located a long distance from the cell body where ubiquitin is synthesized [62]. These results suggest that the effect of USP14 on synapse development and function could be due to a general depletion of synaptic ubiquitin levels, which could indirectly affect ubiquitin modifications at the synapse [58, 62].

Subsequent work has provided additional insight into the specific synaptic changes that underlie the defects in neurotransmitter release at the NMJ of *ax* ^*J*^ mice, including accumulations of phosphorylated neurofilaments, reduced branching and aberrant sprouting of motor neuron terminals, and increased postsynaptic acetylcholine receptor expression combined with immature receptor clusters [62]. Neuron-specific expression of full-length USP14 rescued these developmental defects at the NMJ and the defects in synaptic transmission, including reductions in minifrequency, amplitude, and quantal content observed in the *ax* ^*J*^ mouse [62]. In addition, neuron-specific expression of USP14 rescued the weight loss, reduced viability, and motor deficits observed in *ax* ^*J*^ mice [60]. Importantly, neuron-specific expression of Usp14 also restored cellular monomeric ubiquitin to wild type levels, confirming the role of this proteasome-associated DUB in governing ubiquitin homeostasis and supporting a presynaptic role for USP14 in the ataxia phenotype of the *ax* ^*J*^ mice [60, 62].

Since USP14 has many potential functions in neurons, Wilson and colleagues directly tested a role for USP14 in regulating the abundance of monomeric ubiquitin by expressing ubiquitin itself from a neuronal promoter in the *ax* ^*J*^ mouse. Neuron-specific expression of ubiquitin was able to completely restore ubiquitin in the *ax* ^*J*^ mouse to wild type levels and, impressively, completely rescued the reduced body mass and early postnatal lethality in these animals [67]. Furthermore, neuronally expressed ubiquitin rescued the defects in NMJ development, motor function, and synaptic transmission observed in *ax* ^*J*^ mice [67]. This study elegantly shows that although USP14 may have multiple functions at the proteasome, its role in maintaining cellular levels of monomeric ubiquitin in neurons is central to its function in synapse development and synaptic transmission.

## 4. DUBs That Regulate Known Synaptic Targets

### 4.1. USP14 Regulates GABA_A_ Receptor Synaptic Abundance

In addition to defects in synapse development and function at the NMJ and in the hippocampus, another study found that *ax* ^*J*^ mice exhibit defects in GABA receptor expression in cerebellar Purkinje neurons. Specifically, surface levels of postsynaptic GABA_A_ receptors, as well as the amplitudes of spontaneous inhibitory postsynaptic currents (IPSCs), were increased in Purkinje neurons in *ax* ^*J*^ mice compared to control animals [68]. Colocalization experiments and in vitro binding studies further indicated that USP14 and GABA_A_ receptors are found together at synapses, that the C-terminus of USP14 can interact with the *α*1 loop of the GABA_A_ receptor, and that expression of a GABA receptor peptide that binds USP14 can promote expression of surface GABA_A_ receptors in HEK cells [68]. The authors propose that in addition to its proteasome-associated function to maintain cellular pools of monomeric ubiquitin, USP14 may also have a postsynaptic role in regulating the surface abundance of GABA receptors by specifically deubiquitinating proteins at the plasma membrane or in endocytic vesicles [68]. Since synaptosomes from *ax* ^*J*^ mice have a 40% decrease in ubiquitin conjugates and a 60% decrease in monomeric ubiquitin [62], it will be important to test in future studies whether this general depletion of the free ubiquitin pool indirectly contributes to the effects of USP14 on GABA receptors.

### 4.2. Fat Facets/USP9X Controls Endocytic Protein Abundance to Regulate Presynaptic Function

*Fat facets* (*faf*), the *Drosophila *homolog of mammalian USP9X, was the first DUB shown to play a role in neuronal differentiation, as well as the first DUB for which a synaptic substrate was identified [27, 55]. *Faf *was originally described by Fischer-Vize and colleagues as a gene required for cell fate determination during *Drosophila *photoreceptor development [85]. *Faf *encodes a DUB that genetically and biochemically interacts with *liquid facets* (*Lqf*), the Drosophila homolog of epsin, which is involved in clathrin-mediated endocytosis [86–89]. Further studies suggested that Faf deubiquitinates Lqf to promote endocytosis of the Notch ligand, Delta, during fly eye development [90].

Faf and Lqf have also been shown to function at the *Drosophila *neuromuscular junction (NMJ). DiAntonio et al. found that overexpression of Faf in neurons results in synaptic overgrowth and defects in synaptic transmission [55]. Overexpression of Faf in the developing nervous system induces a dramatic increase in the number of presynaptic boutons, the number of branches and the total area covered by the synapse [55]. Although the size of the NMJ was greatly expanded, neuronal overexpression of *faf* impaired synaptic transmission resulting in a reduction in the quantal content and frequency of evoked and spontaneous excitatory junctional potentials. Interestingly, the reduction in synaptic transmission seen with Faf overexpression is phenocopied by loss of function of the E3 ubiquitin ligase Highwire (*Hiw*). In addition, the defects in synaptic transmission observed in *hiw* mutants are partially suppressed by loss of function *faf* mutants, emphasizing that the balance between ubiquitinating and deubiquitinating activities is critical for normal synaptic function [55]. A subsequent report showed that loss of function *lqf* mutants completely suppress the effects of *faf* overexpression on synaptic bouton number at the NMJ [56], consistent with prior studies indicating that Faf antagonizes the ubiquitination of Lqf [87]. However, unlike overexpression of Faf, overexpression of Lqf did not result in increased branching, suggesting that Faf may have additional synaptic substrates [56]. The effects of Faf were not diminished by mutations in the clathrin adaptin LAP/AP180, which is required for endocytosis of SV components, suggesting that the role of Lqf in synapse development may be independent of SV recycling [56]. The relationship between Hiw and Lqf may be more complex. Unlike what is seen with loss of function *faf *mutants, loss of function *lqf *mutants do not suppress the synaptic overgrowth phenotype of *hiw *mutants, as would be expected if Lqf is an Highwire substrate [56].

In support of a conserved relationship between Faf and Lqf in mammals, USP9X and epsin have been shown to co-immunoprecipitate from rat brain lysates and to colocalize at synapses in brain slices [27]. Interestingly, this study also showed that high potassium-induced depolarization of rat brain synaptosomes results in a large, calcium-dependent decrease in ubiquitin-conjugated proteins, including epsin, suggesting that synaptic activity might stimulate DUB activity [27]. Furthermore, the calcium-dependent deubiquitination of epsin requires USP9X because RNA interference (RNAi)-mediated knock-down of USP9X in HeLa cells results in increased amounts of ubiquitinated and total epsin protein [27].

### 4.3. USP46 Regulates Glutamate Receptor Abundance and GABA-Dependent Behaviors

Several recent studies in worms and mice have uncovered roles for the DUB USP46 in regulating both glutamatergic and GABAergic signaling.

With regard to glutamatergic signaling, work by Kowalski et al. demonstrated that USP-46 regulates the degradation of the glutamate receptor GLR-1 through the MVB/lysosome pathway in *C. elegans* interneurons [69]. Studies in *C. elegans* were the first to show that glutamate neurotransmitter receptors are regulated by ubiquitin. Burbea et al. demonstrated that ubiquitin is directly conjugated to the cytoplasmic tail of the AMPA-type glutamate receptor GLR-1, resulting in its clathrin-mediated endocytosis and subsequent degradation in the lysosome [91, 92]. Further studies showed that mammalian AMPA receptors are also regulated by ubiquitin [93–97], and several ubiquitin ligases have been found either to directly or indirectly regulate glutamate receptor levels at synapses in both invertebrates [98–102] and mammals [94, 95, 97, 103].

Kowalski et al. identified *usp-46 *in an RNAi screen in *C. elegans* for DUBs that regulate the abundance of GLR-1 at synapses in the ventral nerve cord (VNC) [69]. USP46 is a 366 amino acid protein that consists mostly of the catalytic core and is a member of the USP cysteine protease family of DUBs [17]. Mammalian USP46 was first cloned by Quesada, et al. and was shown to exhibit DUB activity towards a model substrate in a bacteria-based assay [104]. Mammalian USP46 is highly homologous (88% homologous) to USP12 [17] and is broadly expressed in a variety of tissues including the brain, heart, and skeletal muscle [104]. In situ hybridization data indicate that USP46 is expressed in several regions of the brain including the hippocampus, amygdala, and cerebellum [70]. The *C. elegans* genome encodes only one of these two highly related DUBs, which is named USP46 [69].

Using quantitative imaging and western blot analyses, Kowalski and colleagues demonstrated that *usp-46 *mutants have reduced levels of GLR-1 receptors in the cell body and processes of interneurons [69]. This reduction in GLR-1 abundance in *usp-46* mutants could be rescued by expression of wild type USP-46, but not by a catalytically-inactive version of USP-46, in interneurons. These data indicate that USP-46 functions specifically in *glr-1*-expressing interneurons to regulate GLR-1 levels in a manner that is dependent on its catalytic activity. Biochemical experiments indicated that the levels of ubiquitinated GLR-1 receptor were increased in *usp-46* mutants and that a nonubiquitinatable version of GLR-1(4KR), where all four cytoplasmic lysine residues are mutated to arginine, is resistant to the effects of *usp-46* mutation. These findings suggest that USP-46 functions to deubiquitinate GLR-1 and to protect the receptor from degradation. In addition, GLR-1 accumulates in the VNC of *usp-46 *mutants when trafficking to the MVB is blocked, suggesting that USP-46 is required to prevent the degradation of ubiquitinated receptors in the MVB/lysosome pathway. Colocalization studies further suggest that USP-46 may function at a RAB5-positive endosome in the cell body and VNC to regulate GLR-1 stability. The authors proposed that USP46 functions at an internal compartment to regulate the pool of receptors available for delivery to the synaptic membrane and that this pool consists of both newly synthesized receptors arriving from the golgi and internalized receptors from the cell surface [69]. Finally, USP-46 regulation of GLR-1 is physiologically relevant, because *usp-46 *mutant worms exhibit defects in glutamate-dependent behaviors (i.e., spontaneous locomotion reversals and a mechanosensory reflex) consistent with decreased glutamatergic signaling.

One question that remained from these studies was the mechanism by which USP-46 activity is regulated in neurons. Biochemical results indicate that although bacterially-expressed recombinant USP46 can interact with GLR-1 from worm extracts, it only exhibits a low level of DUB activity [69]. This result suggests that additional cofactors may be required for full catalytic function. The amino acid sequence of USP-46 offers few clues given the lack of any obvious structural motifs apart from the catalytic domain; however, *C. elegans *USP-46 is closely related to human USP46 and USP12 (60% similarity and 71% identity between *C. elegans *USP-46 and either mammalian USP46 or USP12), and Alan D'Andrea's group found that full activity of USP12 in vitro requires binding with the WD40 repeat-containing proteins, UAF1/WDR48 and WDR20 [105, 106]. Similarly, the catalytic activity of the USP46 homolog in yeast, UBP9, was shown to be dependent on the presence of these WD40 repeat proteins in vivo [107]. Interestingly, UAF1/WDR48 can fully activate the DUB activity of USP1, which regulates the Fanconi anemia DNA repair pathway [108], but only weakly stimulates the catalytic activity of USP46 and USP12 [105, 106, 108], whereas full activation of USP12 requires ternary complex formation with both UAF-1/WDR48 and WDR20 [106].

In a parallel study, Sowa et al. performed an impressive large-scale proteomic study to define the DUB interaction networks for the vast majority of human DUBs and, in so doing, identified several proteins that interacted with both USP12 and USP46 [109]. These interacting proteins included UAF1/WDR48, WDR20, and another WD40-repeat containing protein called DMWD, as well as PHLPP and PHLPPL, two phosphatases that regulate Akt signaling [109]. A subsequent systematic analysis of DUB interactors and subcellular localization in *S. pombe* confirmed the interaction of yeast UBP9/USP46 with BUN62/WDR20 and BUN107/WDR48 [107]. This study also showed that UBP9/USP46 was localized to both the nucleus and several cytoplasmic structures, and that the subcellular localization of UBP9 to the cytoplasmic structures was dependent on BUN107/WDR48 [107]. Together, these reports suggest that the activity and substrate specificity of USP46, and likely other DUBs, can be regulated by interacting proteins.

In neurons, in addition to its role in controlling glutamatergic signaling in worms, two recent reports implicate USP46 in regulating the GABAergic system in mice. Tomida et al. used two behavioral assays, the tail suspension test (TST) and the forced swim test (FST), to monitor depression-like behaviors in mice [70]. These assays subject animals to an inescapable stress and measure the amount of time they are immobile and thus presumably in “behavioral despair.” Antidepressants have been shown to reduce the immobility time supporting the use of these assays as a measure of depression-like behavior. Interestingly, the CS strain of inbred mice exhibit dramatically decreased immobility time in the TST and FST. Tomida et al. used quantitative trait locus (QTL) analysis to map the mutation responsible for this behavior to the *Usp46* gene [70]. Sequencing analyses revealed that the CS mice contain a 3-base pair in frame deletion of a conserved lysine residue in the coding region of the *Usp46 *gene [70]. A subsequent study used a bacteria-based DUB assay to show that this single amino acid deletion reduces but does not eliminate the catalytic activity of USP46 [110]. Importantly, the decreased TST and FST immobility and the defects in the GABA system observed in CS mice could be rescued by expression of *Usp46* from a BAC transgene [70]. To provide further evidence that mutation of *Usp46* was responsible for the antidepressant activity in the TST, Imai et al. generated *Usp46* knock-out mice and showed that these knock-out mice have identical reductions in TST immobility time compared to the CS mice [71]. USP46 appears to affect the GABAergic system because immunohistochemical studies revealed reduced expression of a GABA synthetic enzyme GAD67 in the hippocampus of CS mice [70]. Electrophysiological recordings of CA1 hippocampal neurons from CS mice revealed a small decrease in GABA_A_ receptor-mediated muscimol currents although no changes in mini-IPSC frequency or amplitude were observed [70]. Both of these defects in the GABA system could be rescued by expression of *Usp46* from a BAC transgene [70]. In addition, the decreased TST immobility of the *Usp46* knock-out mice could be corrected by administration of nitrazepam, which increases GABA binding to its receptor, suggesting that *Usp46 *loss of function mice exhibit reduced GABA signaling [71]. These studies implicate USP-46 in regulating the GABA system, although the exact mechanism by which USP46 functions in this pathway awaits further investigation.

Outside of the nervous system, several recent studies have identified diverse roles for USP46 and USP12 in both the nucleus and the cytoplasm and have begun to reveal some of their relevant substrates. Joo et al. demonstrated that USP46 and USP12-containing fractions from HeLa cells can deubiquitinate histones H2A and H2B to control cell fate and gastrulation during *Xenopus *development [111]. In another study, USP46 was shown to function as a tumor suppressor in colon cancer cells by deubiquitinating and stabilizing the phosphatase PHLPP resulting in a downregulation of Akt-mediated cell proliferation and tumorigenesis [112]. Finally, Moretti and colleagues recently described a requirement for USP12-WDR48, but not USP46-WDR48, in promoting the degradation of unactivated Notch receptors in *Drosophila *and in cultured cells via the MVB/lysosome pathway [113]. Although this study supports a role for USP12 in endosomal-lysosomal trafficking, the effect is opposite to that observed in *C. elegans* where USP-46 prevented the degradation of glutamate receptors in the lysosome [69]. Thus, perhaps not surprisingly given the relatively low number of DUBs compared to ubiquitin ligases, the functions of DUBs are diverse and may have different effects depending on the specific substrate and/or cell type being examined.

### 4.4. USP4 Controls the Abundance of the Adenosine G-Protein Coupled Receptor

In addition to regulating the degradation of cytosolic proteins via the proteasome, DUBs are involved in protein quality control in the endoplasmic reticulum (ER) where they regulate the degradation of misfolded or damaged transmembrane proteins via the ER-associated degradation (ERAD) pathway [114]. For example, USP4 was shown to directly bind and deubiquitinate the adenosine A2 (A_2A_) G-protein coupled receptor (GPCR) and prevent its degradation in the ERAD pathway [53]. This study also showed that USP4 promotes A_2A_ receptor abundance on the cell surface because RNAi knock-down of USP4 in HEK293 cells results in decreased levels of surface A_2A_ receptors. Conversely, overexpression of USP4 in hippocampal neurons resulted in a decrease in levels of ubiquitinated A_2A_ receptors and a corresponding increase in the numbers of functional receptors on the cell surface [53]. These effects were specific to A_2A_ receptors, as similar effects of USP4 were not seen for another synaptic GPCR, mGluR5 [53]. Given that a recent study showed that USP4 localizes to the plasma membrane and directly stabilizes surface levels of TGF*β* receptors [115], it will be interesting to explore whether USP4 also influences surface levels of A_2A_ receptor by either antagonizing endocytosis or preventing lysosomal degradation.

## 5. Other DUBs Expressed at Synapses

### 5.1. USP8/UBPY

USP8 (also known as UBPY in humans) plays an important role in a number of cellular processes, including the regulation of receptor tyrosine kinase degradation in the lysosome [15, 20]. USP8 has been shown to have opposing effects on EGF receptor degradation by either directly deubiquitinating receptors to prevent their degradation, or by deubiquitinating ESCRT complex proteins to stabilize them and thus promote EGF receptor degradation [15, 20, 116–119].

Despite the extensive studies on USP8/UBPY in nonneuronal cells, a potential role for this DUB in neurons has been suggested based on several expression and localization studies. USP8/UBPY was originally identified as a protein capable of interacting with and regulating the ubiquitin status of the brain-specific *Ras* guanine nucleotide exchange factor, Ras-GRF1 [120]. Multiple expression analyses revealed that USP8/UBPY is highly expressed in the mouse brain including the hippocampus, hypothalamus, and cerebellum [54, 120]. Subcellular localization studies showed that USP8/UBPY is highly expressed in neurons such as dopaminergic neurons and colocalizes with VAMP proteins, suggesting a potential presynaptic role [54].

### 5.2. USP48/SynUSP

Several recent reports have identified a potential postsynaptic function for the DUB USP48. The rat homolog of this DUB was originally cloned as synUSP from a rat forebrain cDNA library, and in situ hybridization revealed dendritic expression in both cultured cortical neurons and in cortical and hippocampal sections [72]. Additional biochemical analyses revealed that synUSP is enriched in the postsynaptic density and in dendritic lipid raft fractions and exhibits a low level of DUB activity in vitro [72]. The human homolog of synUSP, USP48, was subsequently identified and was also shown to be expressed in the brain [104]. However, although it contains all of the key structural features of the USP enzymes, human USP48 did not exhibit in vitro catalytic activity in an artificial bacterial expression system [104]. Since most DUBs appear to have low intrinsic catalytic activity and the presence of regulatory partners is required for the full enzymatic function of DUBs such as USP1, USP46, and USP12, it will be interesting to study whether other cofactors are required to stimulate USP48 activity in vivo.

## 6. Concluding Remarks

Ubiquitin has emerged as a critical regulator of synapse development and synaptic transmission, and many ubiquitin system components have been identified at the synapse [1, 3, 4]. Mutation of several ubiquitin ligases and DUBs is linked to neurological diseases, including Parkin and UCH-L1 in Parkinson's Disease, Ube3A in Angelman's Syndrome, and USP14 in ataxia [3, 6, 7]. Thus, it is clear that regulation of ubiquitination is important for normal nervous system function. Although we are only just beginning to uncover the function of DUBs at the synapse, these enzymes are attractive candidates for pharmacological intervention. Because ubiquitination of proteins can regulate their localization, activity or degradation, pharmacological inhibition of DUBs could potentially interfere with these various cellular fates of the target proteins. For example, inhibition of a specific DUB could provide a mechanism to downregulate, but not completely eliminate, the activity of a specific target protein that contributes to disease. Additionally, if mutation of a specific ligase contributes to a disease by reducing ligase function, then inhibition of the DUB that counteracts that ligase might alleviate symptoms.

In this review, we have highlighted roles for the small number of DUBs that have been shown to regulate synapse function (Table 1). However, many questions regarding the role of DUBs at the synapse remain: Which specific DUBs function at the synapse to regulate transmission? What are the relevant substrates of DUBs at synapses of different types? Do synaptic DUBs interact with different networks of proteins compared to those observed in nonneuronal cells? Do these interacting proteins regulate DUB catalytic activity, substrate recruitment, or subcellular localization? Does synaptic activity regulate DUB function or localization? Given the importance of ubiquitin in regulating synapse development and function and the large number of ubiquitin ligases and DUBs encoded by the human genome, it is likely that future investigations will identify many more important functions for DUBs and their substrates in synapse biology.

## Figures and Tables

**Table 1:** Summary of DUBs at the synapse.

DUB	Neuronal localization and function	References
UCH family DUBs		

UCH-L1/Ap-Uch	Localizes to dendritic spines and PSD in hippocampal neurons; maintains cellular levels of free ubiquitin by deubiquitinating precursor molecules and stabilizing monomeric ubiquitin; exhibits ubiquitin ligase activity as a dimer; required for normal synaptic structure and function; required for synaptic plasticity in *Aplysia* and mice; implicated in synaptic transmission defects in several neurodegenerative disorders including PD, AD, and *gad *	[26, 33–50]

UCH-L3/Ap-Uch	Required for long-term facilitation in *Aplysia* and working memory in mice	[26, 51]

UCH-L5/UCH37	Associated with the 26S proteasome at synapses; detected in PSDs of hippocampal neurons	[47, 52]

USP family DUBs		

USP4	Binds and deubiquitinates adenosine A2 receptors to prevent their degradation via ER-associated degradation and promotes their surface expression	[53]

USP5	Associated with the 26S proteasome at synapses	[52]

USP7	Associated with the 26S proteasome at synapses	[52]

USP8/UBPY	Highly expressed in brain; colocalizes with presynaptic markers	[54]

USP9x/Fat facets	Deubiquitinates the endocytic protein epsin in *Drosophila* and mammals; regulates presynaptic development and function in *Drosophila *	[27, 55, 56]

USP13	Associated with the 26S proteasome at synapses	[52]

USP14	Localized both pre- and postsynaptically; associated with the 19S regulatory particle of the proteasome; inhibits proteasome-mediated degradation of substrates by trimming ubiquitin chains and maintains cellular levels of free ubiquitin; loss of function mutations in mice result in defects in synapse development at the NMJ, and defects in synaptic transmission at both central and peripheral synapses, as well as ataxia; negatively regulates surface levels of GABA receptors in Purkinje neurons	[57–68]

USP46	Binds and deubiquitinates GLR-1 glutamate receptors to prevent their degradation in the MVB/lysosome pathway in *C. elegans*; Promotes GABA-dependent behaviors in mice	[69–71]

USP48/synUSP	Expressed in dendrites in cortical and hippocampal neurons; Enriched in PSDs and lipid rafts	[72]

Abbreviations: PD: Parkinson's Disease; AD: Alzheimer's Disease; gad: gracile axonal dystrophy; PSD: postsynaptic density.

## Abbreviations

DUB: Deubiquitinating enzyme
USP: Ubiquitin-specific protease
UCH: Ubiquitin C-terminal hydrolase
MVB: Multivesicular body
ESCRT: Endosomal sorting complex required for transport.
